# Response to Commentary on “Sleep Phenotypes, Genetic Susceptibility, and Risk of Obesity in Patients With Type 2 Diabetes: A National Prospective Cohort Study”

**DOI:** 10.1111/1753-0407.70145

**Published:** 2025-08-23

**Authors:** Lei Xi, Juan Shi, Ying Peng, Yifei Zhang, Yanan Cao, Weiqing Wang

**Affiliations:** ^1^ Department of Endocrine and Metabolic Diseases, Shanghai Institute of Endocrine and Metabolic Diseases, National Clinical Research Center for Metabolic Diseases (Shanghai), Ruijin Yangtze River Delta Health Institute, Research Unit of Clinical and Basic Research on Metabolic Diseases of Chinese Academy of Medical Sciences, Wuxi Branch of Ruijin Hospital, Ruijin Hospital Shanghai Jiao Tong University School of Medicine Shanghai China; ^2^ National Center for Translational Medicine (Shanghai), Institute of Translational Medicine Shanghai Jiao Tong University Shanghai China

**Keywords:** genetic risk, obesity, sleep, type 2 diabetes, weight gain

Dear Editor,

We thank the authors for their insightful comments and for recognizing that our manuscript provides valuable details about behavioral and genetic factors affecting the risk of obesity in people with type 2 diabetes (T2D) [1].

Firstly, as mentioned in our article, there may be recall bias based on patient self‐reported sleep duration, and objectively measuring habitual sleep duration using actigraphy can provide more reliable data. However, considering its simplicity, practicality, and correlation with instrument measurement results, patients' self‐reported sleep duration is still internationally recognized and widely used in population‐based studies [2, 3].

Secondly, the relationship between sleep, diabetes and obesity is really complex and challenging. As mentioned by the authors, most exploratory experiments on sleep are temporary, especially for sleep deprivation. There are interactions among sleep, diabetes and obesity that impact cardiovascular and metabolic health, just like the intertwined trio. To avoid the potential impact of sleep related diseases, we excluded patients who reported implausible values of sleep duration (i.e., < 3 or > 12 h/night), use of sleeping aids, or psychiatric medications at baseline. It should be pointed out that the above efforts cannot completely eliminate the potential impact of sleep disorders on our study results, and how to reduce their potential effects is also one of the key considerations in our future research. The Pittsburgh Sleep Quality Index (PSQI) and the Epworth Sleepiness Scale (ESS) are indeed effective tools.

As we mentioned in the article, we acknowledge that “although we have adjusted for several covariates, there are potential confounders that could influence the results, such as dietary habits, physical activity, socioeconomic biases, and glucose‐lowering medications, which we did not include in this study, and further research is needed to strengthen our understanding of these complex associations”, when it comes to both clinical and genetic analysis. In addition, a more complex and precise polygenic risk score (PRS) model for body mass index (BMI) is also under consideration.

In conclusion, we thank the authors for the wise and valuable comments and suggestions. We hope these clarifications address the issues raised and we intend to use more complex and appropriate models to explore their associations in future research.

## Conflicts of Interest

The authors declare no conflicts of interest.
